# *FveTRM5* plays a critical role in regulating fruit shape in woodland strawberry

**DOI:** 10.1093/hr/uhaf199

**Published:** 2025-07-29

**Authors:** Zhenzhen Zheng, Liyang Wang, Qi Gao, Shaoqiang Hu, Chunying Kang

**Affiliations:** National Key Laboratory for Germplasm Innovation & Utilization of Horticultural Crops, Huazhong Agricultural University, Wuhan 430070, China; Hubei Hongshan Laboratory, Wuhan 430070, China; National Key Laboratory for Germplasm Innovation & Utilization of Horticultural Crops, Huazhong Agricultural University, Wuhan 430070, China; Hubei Hongshan Laboratory, Wuhan 430070, China; National Key Laboratory for Germplasm Innovation & Utilization of Horticultural Crops, Huazhong Agricultural University, Wuhan 430070, China; Hubei Hongshan Laboratory, Wuhan 430070, China; National Key Laboratory for Germplasm Innovation & Utilization of Horticultural Crops, Huazhong Agricultural University, Wuhan 430070, China; Hubei Hongshan Laboratory, Wuhan 430070, China; National Key Laboratory for Germplasm Innovation & Utilization of Horticultural Crops, Huazhong Agricultural University, Wuhan 430070, China; Hubei Hongshan Laboratory, Wuhan 430070, China

## Abstract

Cultivated strawberry is a globally important fruit crop with high economic value. Fruit shape is an important aspect of fruit quality and diversity, and a key target in breeding programs; however, few regulatory genes governing fruit shape are known in strawberry. Here, we identified an ethyl methanesulfonate (EMS) ‘round fruit’ (*rf*) mutant that produces round or flat fruits in woodland strawberry. The causal point mutation is located in the second exon of FvH4_2g22810, resulting in a premature termination at amino acid 266. The encoded protein shares a high sequence similarity with TON1 RECRUITING MOTIF 5 (TRM5) in different plant species and was therefore named FveTRM5. Consistently, transforming the *rf* mutant with *FveTRM5pro*:*FveTRM5* restored the wild-type fruit phenotype. *FveTRM5* is ubiquitously expressed in various organs, and the protein localized to microtubules. Overexpression of *FveTRM5* produced elongated organs in both Arabidopsis and woodland strawberry, suggesting a conserved role in different species. Observation of cell shape showed that *FveTRM5* promotes cell elongation and inhibits cell division in the medial-lateral direction in the receptacle. Transcriptome analysis revealed 183 differentially expressed genes (DEGs) in the young receptacles of *rf* and 2976 DEGs in those of *FveTRM5-OE*, including several involved in the auxin and gibberellic acid pathways. In conclusion, our results suggest that FveTRM5 plays an essential role in regulating strawberry fruit shape by influencing cell elongation and cell division, providing an excellent target gene for breeding new fruit shape cultivars.

## Introduction

The shape of a fruit is a key external quality characteristic that strongly influences consumer preference. The organization of floral organs and which part can develop into fruit flesh has evolved differently among species [1, 2]. The diversity of fruit shapes arises from the meristem activity and the growth patterns along the adaxial–abaxial, proximal-distal, and mediolateral axes during ovary and fruit development [1, 3, 4]. Investigating the regulation of fruit shape is essential for the genetic improvement of fruit crops to create new cultivars.

Three gene families are known to play key roles in the control of organ shape in different plant species, including the TONNEAU1 Recruiting Motif proteins (TRMs), the Ovate Family Proteins (OFPs), and the IQ67 domain-containing proteins (IQDs) [5–7]. Mutation or overexpression of TRMs can cause altered shapes of various organs, including seeds/grains/fruits in Arabidopsis [8, 9], rice [10, 11], wheat [12], maize [13], tomato, and cucumber [14, 15]. In the OFP family, OVATE and SlOFP20 in tomato are negative regulators of fruit elongation [14, 16], and *PpOFP1* overexpression due to genomic inversion results in flat fruits in peach [17–19]. The IQD protein SUN1, a calmodulin-binding protein, promotes fruit elongation in tomato [20]. A number of other OFPs or IQDs have also been reported to influence organ shape [21].

Fruit shape is closely related to cell division and cell expansion in different directions. The TRM proteins may affect cell division, cell expansion, or both of them [9, 10, 12, 14, 15]. A common mechanism underlying this biological process is the modulation of the microtubular cytoskeleton. TRMs can recruit TON1 and phosphatase 2A to the microtubule arrays and the preprophase bands to control the direction of cell division [8, 22–25]. TRMs can also interact with OFPs to antagonistically modulate microtubule arrangement [6, 14]. Tomato SlTRM5 localizes to the microtubules, whereas SlOFP20 localizes to the cytoplasm and nucleus; when *SlTRM5* and *SlOFP20* were transiently expressed together in *Nicotiana benthamiana* leaves, SlOFP20 was largely translocated to the microtubules [14]. SlSUN1/IQD12 also localizes to microtubules. Increasing expression of *SlSUN1/IQD12* resulted in long fruits in tomato by changing cell division patterns that is likely associated with altering microtubule structure [26]. In this process, IQDs may interact with calmodulin (CaM) to sense and respond to the calcium signals [27]. The TRM-OFP module may coordinate with IQDs on the microtubular cytoskeleton to regulate organ shape [5, 6].

Cultivated strawberry (*Fragaria* × *ananassa*) is a globally important fruit crop with high economic value. Fruit shape contributes to fruit quality and diversity and is therefore a selected trait in strawberry breeding. Unlike most fruit crops, strawberry flesh develops from the enlarged tip of the stem below the floral organs, known as the receptacle [2]. Both wild and cultivated strawberries have different fruit shapes, ranging from flat to long conical [28, 29]. Fruit shape in strawberries responds to environmental stimuli and endogenous factors, such as auxin and gibberellic acid (GA) levels [30, 31]. Some quantitative trait loci (QTLs) responsible for natural shape variation in strawberry have been identified [32, 33], but few regulatory genes were reported.

Woodland strawberry (*Fragaria vesca*) is a wild diploid species that typically produces small conical fruits. This species is one of the progenitors of the octoploid cultivated strawberry and is primarily used as a model organism for fundamental research in strawberry genetics [34, 35]. In this study, we isolated the fruit shape control gene, *FveTRM5*, from the ethyl methanesulfonate (EMS) mutant ‘round fruit’ (*rf*) in woodland strawberry. Genetic complementation and overexpression assays validated the important functions of *FveTRM5* in promoting organ elongation. We showed that *FveTRM5* regulates fruit shape by influencing both cell elongation and cell division. Our findings provide an excellent target gene for breeding new fruit shape cultivars and exploring fruit shape regulatory mechanisms in strawberry.

## Results

### The *rf* mutant produces round or flat fruits in woodland strawberry

**Figure 1:**
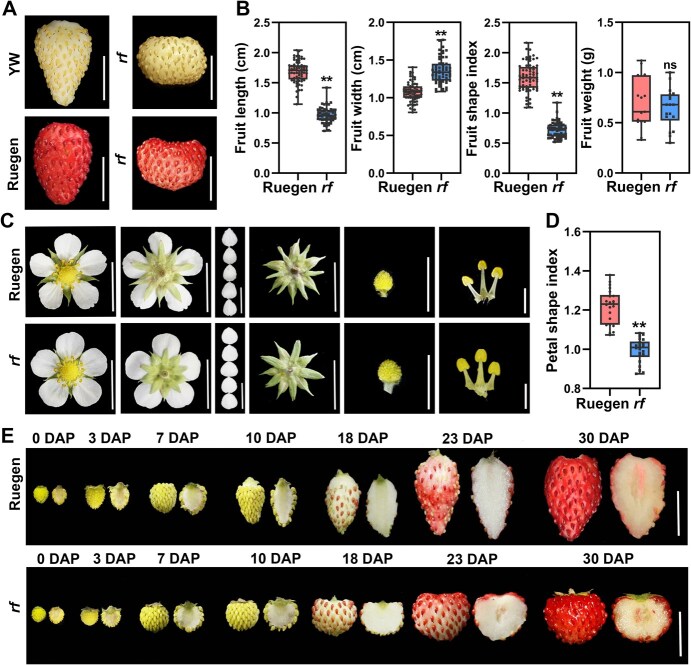
Fruit morphologies in round fruit mutants in woodland strawberry. (A) Mature fruits of wild-type woodland strawberry accessions YW and Ruegen and the *rf* mutant in each background. Scale bars: 1 cm. (B) Fruit width, length, shape index and weight of wild-type Ruegen and *rf*. *n* > 20. (C) Images showing the floral tissues of wild-type Ruegen and the *rf* mutant. Scale bars: 2 mm for stamens and 1 cm for others. (D) Petal shape index of wild-type Ruegen and the *rf* mutant. *n* > 20. (E) Fruits of wild-type Ruegen and *rf* at different developmental stages from anthesis to ripening. DAP, day after pollination. Scale bars: 1 cm. For statistical analysis, data are the mean ± SD; ^**^P < 0.01; ns, not significant, Student’s *t* test.

To isolate genes regulating strawberry fruit shape, we characterized a ‘round fruit’ (*rf*) mutant from the ethyl methanesulfonate (EMS)-treated population of the woodland strawberry variety Yellow Wonder (YW, white-fruited) (Fig. 1A). To better observe fruit development during ripening, we crossed this mutant with the red-fruited accession Ruegen to generate a red-fruited *rf* mutant (Fig. S1A). This hybrid acquired a functional *MYB10* gene from Ruegen, a master regulator of fruit pigmentation in strawberry [36]. The wild-type fruits were conical, whereas the *rf* fruits were nearly round or flat (Fig. 1A). Fruit measurements showed a significant reduction in fruit length and a significant increase in fruit width, resulting in a significantly reduced fruit shape index (length/width) in *rf* compared to wild type, but no difference in fruit weight (Fig. 1B). Other *rf* organs, such as petals, sepals, receptacles, stamens, and leaves, were also shorter or rounder than the wild type (Fig. 1C and D; Fig. S1B and C). Further observation showed that the *rf* receptacle was always shorter than the wild type from anthesis to fruit ripening (Fig. 1E). These results indicate that the *RF* gene is important in regulating the shape of both fruit and other organs in woodland strawberry.

### *FveTRM5* is the causal gene of the *rf* mutant

**Figure 2:**
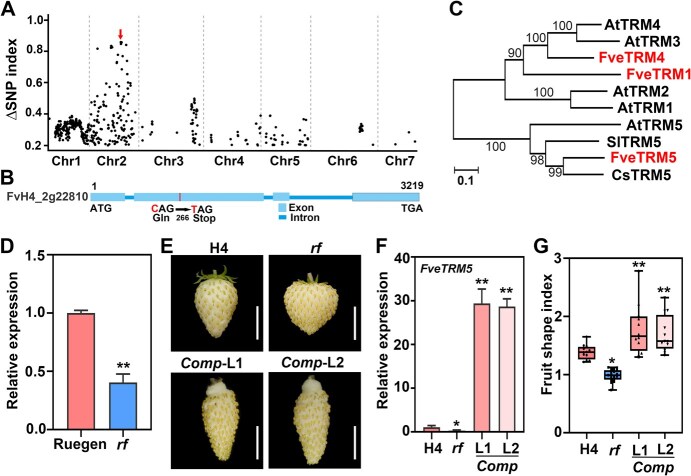
Isolation and characterization of *FveTRM5* in woodland strawberry. (A) Diagram showing the SNPs associated with *rf* along the chromosomes. The X axis represents the chromosomes in woodland strawberry. The Y axis indicates the SNP index differences between the wild type and *rf* mutant pools. The arrow indicates the linked peak on chromosome 2. (B) Diagram showing the candidate causal mutation in FvH4_2g22810 for *rf*. The red letters indicate the candidate point mutation. (C) Phylogenetic tree of the TRM proteins in woodland strawberry (Fve), Arabidopsis (At), tomato (Sl), and cucumber (Cs) in the AtTRM1–5 subclade using full-length sequences. Bootstrap values at the nodes were generated from 1000 replicates. The branch length indicates the number of substitutions per site. (D) Relative expression level of *FveTRM5* in wild-type Ruegen and *rf* fruits (7 DAP) examined by RT-qPCR. *n* = 3. (E) Images showing the mature fruits of wild-type H4, *rf*, and two complementation lines (*Comp*-L1 and *Comp*-L2) in woodland strawberry. Scale bars: 1 cm. (F) Relative expression level of *FveTRM5* in wild-type H4, *rf*, *Comp*-L1 and *Comp*-L2 leaves examined by RT-qPCR. *n* = 3. (G) Fruit shape index of H4, *rf*, *Comp*-L1 and *Comp*-L2. *n* > 10. For statistical analysis, data are the mean ± SD; ^*^, *P* < 0.05; ^**^, *P* < 0.01; Student's *t* test.

To identify the causal gene, the *rf* mutant was crossed with the wild-type woodland strawberry accession Ruegen, resulting in F_1_ progeny that all exhibited the wild-type phenotype. These F_1_ plants were then self-pollinated to produce a segregating F_2_ population. In this F_2_ population, 81 plants produced conical fruits, and 27 plants produced round fruits, fitting a 3:1 ratio (χ^2^ = 0). This suggests that a monogenic recessive mutation is responsible for the *rf* phenotype. Through bulk genome resequencing and subsequent data analysis, a strongly associated peak was detected on chromosome 2 (Fig. 2A). The candidate SNPs should pass the following criteria: (i) G to A or C to T transition; (ii) 100% homozygous in the mutant pool, less than 50% in the WT pool; (iii) located in the coding sequence and causing nonsynonymous mutations in the protein [37]. After filtering, seven candidates were identified in the region from 16.48 to 20.03 Mb (Table S1). One of the candidates is the SNP (C to T) in the second exon of FvH4_2g22810 (annotation ver. 4), which causes premature translation termination at amino acid 266 (Fig. 2B). This was further confirmed by Sanger sequencing of 52 individual F_2_ mutants, each of which was homozygous for the FvH4_2g22810 mutation, suggesting complete co-segregation of the mutation with the fruit shape phenotype. The protein sequence of FvH4_2g22810 shared 35.3% sequence similarity with Arabidopsis TON1 RECRUITING MOTIF 5 (AtTRM5), 52.5% similarity with tomato SlTRM5, and 60.1% similarity with cucumber CsTRM5 [8,14,15]. Therefore, this gene was named *FveTRM5*. We found a total of 18 TRM family members in the woodland strawberry genome with distinct expression patterns (Fig. S2A and B). The AtTRM1–5 subclade contains three woodland strawberry genes, including *FveTRM1* (FvH4_6g46710), *FveTRM4* (FvH4_5g13690) and *FveTRM5* (Fig. 2C). When examined by RT-qPCR, *FveTRM5* was significantly downregulated in the *rf* fruits at 7 DAP (days after pollination) compared to wild type (Fig. 2D), suggesting nonsense-mediated RNA decay.

To validate the gene identity, *FveTRM5pro*:*FveTRM5* was generated and stably transformed into the wild-type Hawaii 4 (H4, white-fruited) for higher transformation efficiency. We obtained a total of 28 transgenic lines with either wild-type or elongated fruits in the T_0_ generation. We selected 2 T_0_ lines (L1 and L2) with elongated fruits to cross with the *rf* mutant. In the F_2_ generation, the transgenic plants with the homozygous *rf* mutation were identified and named *Comp*-L1 and *Comp*-L2 (Fig. 2E). According to RT-qPCR, the expression level of *FveTRM5* in *Comp*-L1 and *Comp*-L2 was 28 to 30 times higher than in the wild type (Fig. 2F). The fruit shape index of *Comp*-L1 and *Comp*-L2 was significantly higher than that of *rf* and wild type (Fig. 2G), indicating that *FveTRM5* could rescue the fruit shape defect in *rf*. These results indicate that *FveTRM5* is the causal gene of the *rf* mutant.

### Expression pattern of *FveTRM5* and subcellular localization of the encoded protein

**Figure 3:**
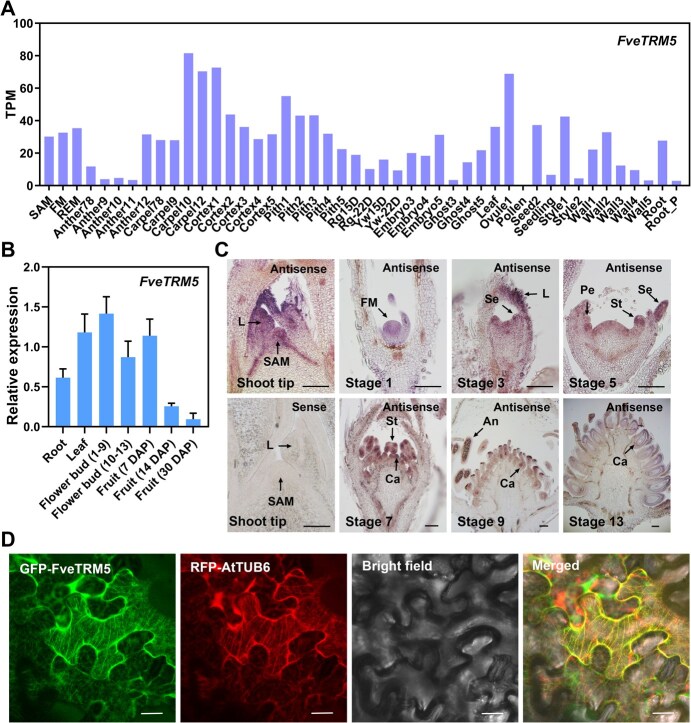
Expression pattern of *FveTRM5* and its protein subcellular localization. (A) Expression pattern of *FveTRM5* in woodland strawberry as indicated by transcripts per million (TPM) values obtained from the transcriptome database [38]. (B) Relative expression levels of *FveTRM5* in the tissues of wild-type Ruegen examined by RT-qPCR. *n* = 3. DAP, days after pollination. (C) Expression pattern of *FveTRM5* in longitudinal sections of wild-type shoot tips and flower buds at different stages by RNA *in situ* hybridization. SAM, shoot apical meristem; L, leaf; FM, floral meristem; Se, sepal; St, stamen; Pe, petal; Ca, carpel; An, anther. Scale bars: 100 μm. (D) Subcellular localization of GFP-FveTRM5 in *N. benthamiana* leaves. Green indicates GFP fluorescence, and red indicates RFP fluorescence of the microtubule marker RFP-AtTUB6. Scale bars: 10 μm.

Based on the transcriptome database of woodland strawberry [38], *FveTRM5* exhibits broad expression across various floral and fruit tissues, including flower meristems (FM), carpels, embryos, and receptacles (cortex and pith), as well as shoot apical meristems (SAM), leaves and roots (Fig. 3A). When examined by RT-qPCR, *FveTRM5* expression was indeed higher in leaves, flower buds and young fruits, moderate in roots, but strongly decreased in mature fruits at 30 DAP (Fig. 3B). We further examined the expression pattern of *FveTRM5* in shoot tips and floral buds by RNA *in situ* hybridization. Flower stages were determined as previously described [39]. Strong signals of *FveTRM5* were detected in the SAM, leaf primordia, FM, receptacle meristem and all the floral organs at flower stages 1 to 13, no signal was detected by hybridization with the *FveTRM5* sense probe (Fig. 3C).

Previous studies have shown that some TRM proteins co-localize with microtubules, a major component of the cytoskeleton [14]. To test this possibility, GFP-FveTRM5 and the microtubule marker RFP-AtTUB6 were simultaneously infiltrated into the *N. benthamiana* leaves for transient expression. The RFP-AtTUB6 signals were shown as lines, indicating the localization of microtubules (Fig. 3D). GFP-FveTRM5 signals colocalized with RFP-AtTUB6 on microtubules (Fig. 3D). This localization is similar to that of SlTRM5, which has been implicated in fruit shape regulation [14, 40].

### Overexpression of FveTRM5 results in elongated organs in Arabidopsis and woodland strawberry

To determine the function of *FveTRM5*, *FveTRM*5 under the control of the 35S promoter was first introduced into WT Arabidopsis. Twenty-five independent *FveTRM5*-OE lines were obtained in the T_1_ generation with much narrower and longer leaves (Fig. S3A). *FveTRM5* expression was confirmed in two transgenic Arabidopsis lines (Fig. S3B). In comparison to the wild type, the overexpression of *FveTRM5* resulted in sufficiently elongated organs, including petals, stamens, gynoecia, siliques, and seeds (Fig. S3C and D). These results suggest that *FveTRM5* could promote organ elongation in alternative species.

**Figure 4:**
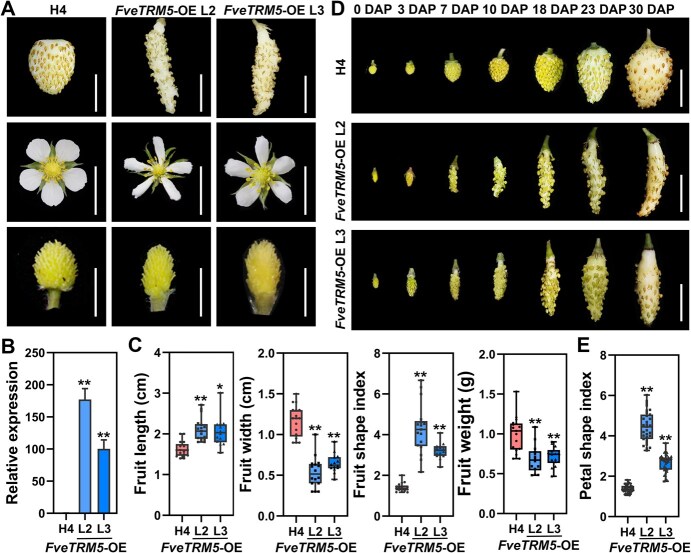
Phenotypic characterization of the *FveTRM5-OE* transgenic lines in woodland strawberry. (A) Images showing the fruits and floral organs of two *FveTRM5*-OE transgenic lines (L2 and L3) in woodland strawberry. Scale bars: 1 cm. (B) Relative expression levels of *FveTRM5* in the leaves of wild-type H4 and *FveTRM5*-OE (L2 and L3) examined by RT-qPCR. *n* = 3. (C) Fruit length, width, shape index and weight of H4 and *FveTRM5*-OE (L2 and L3). *n* > 15. (D) Fruits of wild type H4 and two independent *FveTRM5*-OE lines (L2 and L3) at different developmental stages from anthesis to ripening. DAP, days after pollination. Scale bars: 1 cm. (E) Petal shape index of H4 and *FveTRM5*-OE (L2 and L3). *n* > 20. In (B), (C), and (E), data are presented as mean ± standard deviation (SD). Statistical significance was determined using Student’s *t*-test (^*^P < 0.05, ^**^P < 0.01).

To further validate the function of *FveTRM5* in strawberry, the *FveTRM5*-overexpression construct was also stably transformed into the wild-type woodland strawberry strain H4. We obtained 10 independent *FveTRM5* overexpressing lines. Two lines (*FveTRM5*-OE L2 and L3) were carefully characterized at T_0_. Both lines produced much thinner mature fruits and floral organs, such as petals and receptacles (Fig. 4A). RT-qPCR confirmed that *FveTRM5* was expressed at 177-fold and 100-fold higher levels in these two lines than in the wild type (Fig. 4B). Fruit shape measurements showed that fruit length was significantly greater and fruit width was significantly smaller in *FveTRM5*-OE than the wild type, resulting in a higher fruit shape index (Fig. 4C). It should be noted that the fruit weight also decreased significantly in *FveTRM5*-OE, mainly due to the extreme reduction in fruit width. The change in fruit shape in *FveTRM5*-OE started at anthesis and remained the same until ripening (Fig. 4D). *FveTRM5*-OE also showed an increase in the petal shape index (length/width) (Fig. 4E). In addition, *FveTRM5* overexpression resulted in narrower leaves (Fig. S4A–C). These results support that *FveTRM5* is an important regulator of organ shape in woodland strawberry and may exert similar phenotypes in other plants.

### FveTRM5 regulates cell expansion and cell division in strawberry fruit

**Figure 5:**
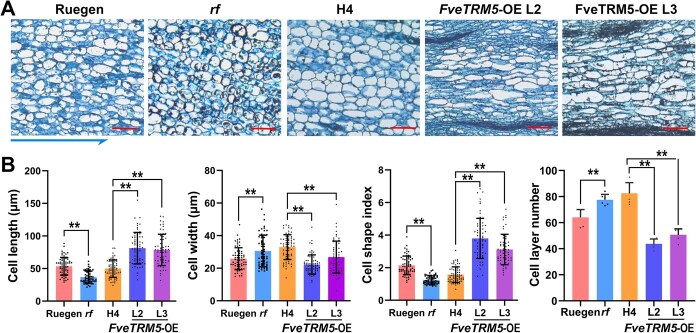
Changes in cell morphology in different materials of FveTRM5. (A) Images showing cell shapes in the receptacle of wild-type Ruegen and H4, *rf*, and *FveTRM5-OE* (L2 and L3) at anthesis. The arrow at the bottom indicates the longitudinal direction of the fruit. Scale bars: 100 μm. (B) Cell shape and number of cell layers in the transverse direction at the widest part of wild-type Ruegen and H4, *rf*, and *FveTRM5-OE* (L2 and L3) receptacles. For statistical analysis, data are the mean ± SD; ^**^, *P* < 0.01; student’s *t*-test. *n* = 60 for cell shapes and 6 for cell layers.

As the change in fruit shape remains the same throughout development, the cell morphology in the receptacles of *rf*, *FveTRM5*-OE and the wild types (Ruegen and H4) at anthesis was examined by paraffin sectioning. Initial observations showed that the receptacle cells appeared shorter and smaller in *rf* and longer in *FveTRM5*-OE (Fig. 5A). Cell measurements showed that *rf* receptacle cells were significantly shorter than wild type, whereas *FveTRM5*-OE receptacle cells were significantly longer than wild type (Fig. 5B), suggesting that FveTRM5 positively regulates cell elongation in the longitudinal direction. In contrast, cell width was significantly decreased in *rf* and the two *FveTRM5*-OE transgenic lines, resulting in the significant change of cell shape index. In addition, there were more cell layers in *rf* and fewer cell layers in *FveTRM5*-OE in the transverse direction, suggesting that *FveTRM5* could inhibit cell division in the medial-lateral direction of the receptacle (Fig. 5B). These results indicate that *FveTRM5* regulates strawberry fruit shape by controlling both cell expansion and cell division.

### Transcriptome profiling of young fruit receptacles in the *rf* mutant and *FveTRM5*-OE lines

**Figure 6:**
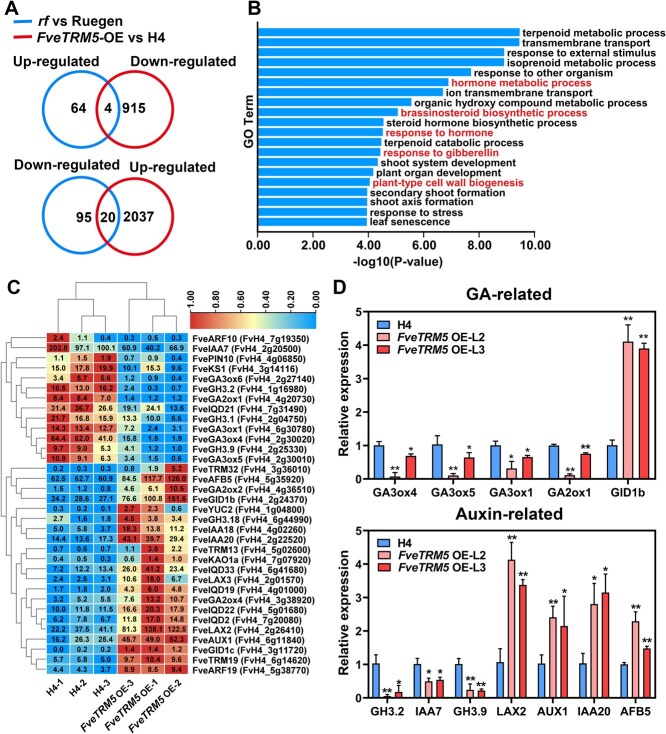
Transcriptome analysis of fruit receptacles of *rf* and *FveTRM5*-OE at 7–10 DAP. (A) Venn diagram showing the differentially expressed genes (DEGs) in fruit receptacles of *rf* compared to Ruegen and of *FveTRM5*-OE compared to H4 at 7–10 DAP (days after pollination). (B) Top 20 enriched GO terms (corrected *P* value < 0.01) among the DEGs in fruit receptacles of *FveTRM5*-OE compared to H4 at 7–10 DAP. (C) Heatmap showing the expression levels of DEGs in auxin and GA pathways and in the *IQD* and *TRM* gene families. The number in each box indicates the average TPM values. Color scale indicates the normalized values among samples for each gene. (D) Expression levels of the selected auxin and GA related genes in fruit receptacles of *FveTRM5*-OE compared to H4 by RT-qPCR. Data are presented as mean ± standard deviation (SD) (*n* = 3). ^*^, *P* < 0.05; ^**^, *P* < 0.01; Student’s *t*-test.

To understand the transcriptome changes in the fruits of different *FveTRM5* genotypes, RNAseq was performed for the fruit receptacles at 7 to 10 DAP in Ruegen, the *rf* mutant, H4 and *FveTRM5*-OE with three biological replicates. As a result, a total of 40 to 56 million clean reads were obtained (Table S2). Pairwise comparisons were used to determine differentially expressed genes (DEGs), with thresholds set at a fold change >1.5, *P* value <0.05, and the maximum expression level of TPM > 1. When *rf* was compared with Ruegen, 68 genes are significantly upregulated, and 115 genes are significantly decreased in *rf* (Table S3). When *FveTRM5*-OE was compared with H4, 2057 genes are significantly upregulated, and 919 genes are significantly downregulated (Table S4). Among them, four genes were up-regulated in *rf* and simultaneously down-regulated in *FveTRM5*-OE; 20 genes were both down-regulated in *rf* and up-regulated in *FveTRM5*-OE (Fig. 6A). As the *rf* mutant has few DEGs, we paid more attention to the DEGs in *FveTRM5*-OE. These genes (2976 in total) showed several enriched gene ontology (GO) terms, including hormone metabolic processes, response to gibberellin and plant-type cell wall biogenesis (Fig. 6B).

We found that *FveTRM5* has an average TPM of 27.6 in H4 and of 1059.5 in *FveTRM5*-OE (Table S4), confirming its high expression in the transgenic lines. In addition, expression levels of five IQD genes and three TRM genes are significantly changed in *FveTRM5*-OE (Fig. 6C). It is known that higher GA levels lead to longer fruit, while increased auxin promotes rounder fruit in strawberry [30]. We found that 11 GA-pathway genes and 15 auxin-pathway genes are differentially expressed in *FveTRM5*-OE compared to H4 (Fig. 6C), consistent with the essential roles of GA and auxin in regulating strawberry fruit shape. To confirm these results, the GA- and auxin-pathway genes with higher expression levels were examined by RT-qPCR in fruit receptacles. Four GA biosynthesis genes (*GA3ox1*, *GA3ox4*, *GA3ox5* and *GA2ox1*) are significantly downregulated, while the GA receptor gene *GID1b* is significantly upregulated in fruit receptacles of *FveTRM5*-OE (Fig. 6D). In the auxin pathway, three genes (*GH3.2*, *GH3.9* and *IAA7*) show reduced expression levels, whereas four genes (*LAX2*, *AUX1*, *IAA20*, and *AFB5*) are significantly induced in *FveTRM5*-OE (Fig. 6D). These data are consistent with the RNAseq results. Of note, several GA genes show smaller changes in L3 than L2, aligning with the less severe fruit phenotype observed in L3. These results suggest that *FveTRM5* overexpression may interfere with the gene network involving hormone pathways and microtubule organization.

## Discussion

Cultivated strawberries produce fruits with a wide variety of shapes, but those with round fruits are relatively rare. In this study, we identified an artificially induced mutant called ‘round fruit’ (*rf*) in woodland strawberry. Gene cloning and genetic analysis revealed that *FveTRM5* is the causal gene for this mutant. Overexpression of *FveTRM5* resulted in elongated organs in both woodland strawberry and Arabidopsis, suggesting a conserved role in different species. Furthermore, *FveTRM5* can inhibit transverse cell division and promote longitudinal cell elongation in the fruit receptacle. This work provides compelling evidence for the role of *FveTRM5* in fruit shape control and makes it a strong candidate for fruit shape manipulation in strawberry and other fruit crops.

The TRM proteins in the AtTRM1–5 subclade promote elongation of fruits as well as other organs across various plant species [9–11, 14, 15]. There are three members in this subclade in the woodland strawberry genome (Fig. 2C). In this study, we have demonstrated the important functions of *FveTRM5* in fruit shape regulation. It remains to be tested whether *FveTRM1* and *FveTRM4* have similar or redundant functions. This possibility is supported by the tomato mutants of *SlTRM3/4* and *SlTRM5*, which show additive effects in shortening the *ovate Slofp20* fruits [40]. In addition, mutations in *SlTRM19* and *SlTRM17/20a* from another subclade could enhance fruit elongation in the *ovate Slofp20* double mutant, suggesting that the TRM proteins from different subclades may also have redundant or opposing functions. Our transcriptome data shows that *FveTRM13* (FvH4_5g02600), *FveTRM19* (FvH4_6g14620) and *FveTRM32* (FvH4_3g36010) are significantly upregulated in *FveTRM5*-OE compared to H4 (Fig. 6C). Although these three *TRM* genes exhibit relatively low expression levels in this dataset, their potential roles in fruit shape regulation remain to be investigated. In octoploid cultivated strawberry, the homologs of *FveTRM5* and other *TRM* members should be further characterized, as they may exhibit differential expression or even absence across different subgenomes.

Some TRM proteins promote organ elongation by stimulating cell division along the longitudinal axis while suppressing it in the transverse direction [9, 14, 15]. Our results showed that *FveTRM5* not only inhibited cell division in the transverse direction but also decreased cell width and increased cell length, a result of anisotropic cell expansion (Fig. 5). Although TRM proteins can be localized on microtubules or in the cytosol [8], SlTRM5 is particularly localized on microtubules [14, 40]. FveTRM5 also localizes to microtubules, similar to SlTRM5. The TRM proteins often interact with OFPs to regulate organ shape [6, 14]. Thus, protein interaction assays may help to find the FveOFPs involved in the fruit shape regulation. RNAseq analysis reveals that *FveOFP1* (FvH4_3g40310) is significantly upregulated in the *rf* mutant (Table S3), suggesting that it is a strong candidate for interaction with *FveTRM5*.

Fruit shape is regulated by plant hormones like auxin and GA, along with other external or internal factors. In tomato, whole-plant application of exogenous auxin before anthesis results in longer ovaries and fruits, with an increased number of cells along the longitudinal axis and enlarged cells in most tissues of the ovary [41]. GA_3_ treatment in tomato results in elongated fruits, whereas application of paclobutrazol, a GA inhibitor, results in more flattened fruits [42, 43]. In woodland strawberry, auxin application results in rounder fruits, whereas GA treatment leads to thinner fruits [30]. We found that the expression levels of 15 auxin genes and 11 GA genes are significantly changed in fruit receptacles of *FveTRM5*-OE compared to those of H4 (Fig. 6C). These results highlight the crucial roles of auxin and GA in strawberry fruit shape regulation. According to the *FveTRM5*-OE phenotypes, we speculate that GA levels and/or GA signal transduction are enhanced, whereas auxin levels and/or signaling are decreased. However, how *FveTRM5* overexpression affects GA and auxin pathways is still enigmatic. The hormone pathways are known to cross-talk with the *OFP* and *IQD* family genes [6, 41, 44, 45], which may indirectly interfere with TRM functions. In addition to hormones, mutation of the red photoreceptor FvePhyB results in fruits with higher shape index [46]. Some upstream regulators of the *TRM* genes have been reported. For example, the bHLH transcription factors Leaf-related Protein 1 (LP1) and LP2 can directly regulate the expression levels of *AtTRM1* and *AtTRM2* to induce longitudinal cell elongation in Arabidopsis [47]. OsSPL16 can directly bind to the *GW7* (ortholog of *AtTRM1*) promoter to inhibit its expression in rice [10]. The mechanisms by which hormones and light signaling regulate *FveTRM5* expression and function warrant further investigation.

In conclusion, we have reported a key player controlling strawberry fruit shape, FveTRM5, which influences cell division and cell elongation in fruit tissues. Based on the genetic studies, we believe that it is possible to knock out *FveTRM5* to produce round fruits. However, *FveTRM5* cannot be overexpressed to high levels as this will reduce the biomass. These findings can provide a theoretical basis for breeding new fruit shape cultivars in strawberry and enrich our knowledge of fruit shape control in fruit crops.

## Materials and methods

### Plant materials and growth conditions

Three *F. vesca* varieties, Yellow Wonder (YW, white-fruited), Ruegen (red-fruited) and Hawaii 4 (H4, white-fruited), were used as the WT in this study. All plants were cultivated in a growth room at 26°C under a 16-hour light/8-hour dark cycle, with a light intensity of 100 μmol m^−2^ s^−1^. To perform EMS mutagenesis, seeds were treated with 0.4% EMS (Sigma, #M0880) for 8 hours at room temperature under shaking. Mutants were isolated from the M_2_ generation.

### Phenotypic analysis

For each genotype, samples from three to five plants (including flowers or fruits) were examined. The length and width of each tissue were determined using ImageJ software, and the shape index was derived as the length-to-width ratio. Three fruits at 0 DAP of Ruegen, H4, *rf* and *FveTRM5*-OE were used for paraffin sectioning. Immediately after collection, the samples were immersed in FAA fixative (a blend of 5% acetic acid, 5% formaldehyde, 70% ethanol). Paraffin embedding and sectioning were performed as previously described [48]. The section thickness was 10 μm for each fruit. For cell shape analysis, 20 cells in the pith with clear shapes were selected from one section of each fruit, resulting in 60 cells for each genotype. For cell number analysis, the cells in the widest part of the receptacle were counted over the entire receptacle from two sections of each fruit, resulting in 6 samples for each genotype.

### Gene cloning of the *rf* mutant

The *rf* mutant was crossed with wild-type Ruegen to generate an F_2_ population. Young leaves were collected in equal quantities from 11 F_2_ mutant and 20 F_2_ wild type plants and then pooled together. The CTAB method was used to isolate the genomic DNA. Sequencing was carried out on the Illumina HiSeq X Ten platform (Novogene, Beijing), followed by analysis using established protocols [37]. The raw reads from the two pools were mapped to the reference genome of woodland strawberry (version 4) using BWA-MEM with default parameters [49, 50]. The SAM file was further transformed into a sorted bam file and duplicate reads were removed using SAMtools [51]. SNP calling was performed with GATK HaplotypeCaller [52] using the parameters ‘–min-base-quality-score 20; –minimum-mapping-quality 20’. Subsequent filtering was applied with the following criteria: QD < 2.0, FS > 60, MQ < 40, MQRankSum < −12.5, ReadPosRankSum < −8.0, and clustering settings of ‘-cluster 2 -window 20’. Only the G to A or C to T site was retained for further analysis. The ∆SNP index was initially computed by determining the difference in SNP frequency between the two pools, followed by a sliding window approach (300-kb window size, 100-kb step size) to derive the mean ∆SNP index. SNPs located in the CDS region were extracted using BEDTools [53]. The candidate SNP was validated in individual F_2_ mutants by PCR amplification and Sanger sequencing.

### Phylogenetic analysis

The FveTRM proteins in woodland strawberry were identified using the Dicots PLAZA 5.0 database accessible at https://bioinformatics.psb.ugent.be/plaza. Other protein sequences were obtained from TAIR for Arabidopsis (Arabidopsis.org), Sol Genomics Network for tomato (solgenomics.net), and Cucurbit Genomics Data for cucumber (cucurbit genomics.org). The phylogenetic tree was generated in MEGA7 using the neighbor-joining method with 1000 bootstrap replicates and was presented as an unrooted tree.

### Plasmid construction

Genomic DNA or total RNA was extracted from fruits or young leaves of Ruegen and used for gene amplification. For overexpression in woodland strawberry, the full-length coding sequence of *FveTRM5* was cloned into pENTR1A and then inserted into the destination vector pK7WG2D. For overexpression in Arabidopsis, the full-length coding sequence of *FveTRM5* was inserted into pRI101 at the *Sal*I and *BamH*I site in fusion with GFP using the ClonExpress II One Step Cloning Kit (Vazyme, C112–01). For *FveTRM5pro*:*FveTRM5*, a 3219 bp genomic fragment of *FveTRM5* (including exons and introns), along with 304 bp upstream of the translation start site and 999 bp downstream of the stop codon, was cloned into the destination vector pCambia1300 using the *Sal*I and *Bam*HI restriction sites. To determine subcellular localization, the *FveTRM5* coding sequence was inserted into the pH7LIC5.0 vector through *Sal*I and *Stu*I restriction sites, creating an N-terminal GFP fusion using the aforementioned kit. Table S5 shows the primers.

### Woodland strawberry transformation

Transformation in woodland strawberry was performed as previously described [54]. The overexpression and complementation constructs were transformed separately into woodland strawberry variety H4. During the transformation process, callus was induced from leaf strips on the induction medium (MS, 0.3 mg l^−1^ indole-3-butyric acid (IBA), 3.4 mg l^−1^ 6-benzylaminopurine (6-BA), 2% sucrose, 0.7% agar, pH 5.8). The tissues were co-cultured for 1 hour with Agrobacterium GV3101 carrying each construct in a co-cultivation buffer (MS, 2% sucrose, 500 mg l^−1^ acetosyringone) under gentle shaking. Subsequently, they were transferred to induction medium and kept in darkness for 4 days. After washing, the tissues were transferred to fresh induction medium supplemented with 250 mg l^−1^ timentin and 250 mg l^−1^ carbenicillin, followed by a 7-day dark incubation period. Afterwards, they were cultured under light and subcultured every 14 to 30 days until shoot emergence. The resulting shoots were transferred to rooting medium (1/2 MS, 0.1 mg l^−1^ IBA, 250 mg l^−1^ carbenicillin, 250 mg l^−1^ timentin, 2% glucose, 0.7% agar, pH 5.8). Transgenic calli and regenerated plants were screened using either 10 mg l^−1^ kanamycin or 4 mg l^−1^ hygromycin, along with GFP fluorescence (when applicable), observed under a fluorescence dissecting microscope (MZX81, Microshot Technology Ltd, Guangzhou, China).

### Arabidopsis transformation

The floral-dip method was employed to transform Arabidopsis Col-0 with *Agrobacterium tumefaciens* GV3101. T_1_ transgenic lines were selected on half-strength MS (M5524, Sigma) containing 100 mg l^−1^ kanamycin.

### Subcellular localization analysis

The abaxial side of *N. benthamiana* leaves was infiltrated with *A. tumefaciens* GV3101 cells carrying the construct and p19 in a 1:1 ratio. The *35S*:*RFP-AtTUA5* construct was co-infiltrated as a microtubule marker [55]. The *Agrobacterium* cells were collected and then suspended in a buffer (10 mM MES, 10 mM MgCl_2_, 150 mM acetosyringone, pH 5.6) and adjusted to an OD_600_ of 0.2. The mixtures were kept at room temperature for 2 hours without shaking prior to infiltration. Fluorescence signals were acquired 4 days after infiltration using a Leica TCS SP8 inverted microscope. The excitation wavelengths used were 488 nm (for GFP) and 552 nm (for RFP), with emission collected at 505 to 550 nm (GFP) and 590 to 640 nm (RFP).

### RT-qPCR

Total RNA was isolated with the HiPure Plant RNA Mini Kit (Cat#R4151, Magen, Guangzhou, China) and then reverse-transcribed into cDNA using HiScript III RT SuperMix (Cat#R323, Vazyme, Nanjing, China). Quantitative PCR was carried out with the PerfectStart® Green qPCR SuperMix (Cat#AQ601, TransGen, Beijing, China). Gene expression level was calculated using the 2^−ΔΔCT^ method. FvH4_1g05910 served as the reference gene. Each sample was analyzed in triplicate, with three technical replicates performed per biological replicate. Primers are listed in Table S5.

### RNA *in situ* hybridization

Wild-type H4 shoot tips and flower buds at various developmental stages were collected and fixed in 50% FAA solution at 4°C. A 200 bp segment (positions 215–414 in the coding sequence) of the *FveTRM5* gene was cloned into the pGEM-T vector using the *Nco*I and *Sal*I restriction sites. DIG-labeled RNA probes were synthesized via *in vitro* transcription using the DIG RNA Labeling Kit (Roche, Cat#11175025910). The hybridization signals were visualized with the DIG Nucleic Acid Detection Kit (Roche, Cat#11175041910). The slides were incubated for 18 hours in a dark, humid container. After stopping the reaction, they were stored in 1× TE buffer. Images were acquired using a Leica DM6B microscope. The primer sequences can be found in Table S5.

### Transcriptome profiling

Total RNA was extracted from the fruit receptacles of *rf*, Ruegen, *FveTRM5*-OE and H4 at 7–10 DAP using the HiPure Plant RNA Mini Kit (Magen, Guangzhou, China, R4151–02). The *FveTRM5*-OE samples were collected for L2 and L3 lines and mixed. Three biological replicates were prepared for each sample. The sequencing procedure was performed using an Illumina Nova Seq platform (Novogene, Beijing). The woodland strawberry genome (version 4) served as the reference. The raw reads were first trimmed (9 bp from the 5′ end) with Trimmomatic and subsequently mapped to the genome using Hisat2 [56, 57]. Gene expression levels were calculated as transcripts per million (TPM) reads using TBtools [58]. Gene expression differences were analyzed by using DESeq2 (R package), with cutoff values of fold change >1.5, *P* value < 0.05, and the maximum TPM > 1 [59]. GO enrichment and heatmap analysis was performed using TBtools software.

### Statistical analyses

Statistical analysis was conducted using GraphPad Prism 8. For pairwise comparisons, Student’s *t*-test was applied (ns, not significant; ^*^P < 0.05; ^**^P < 0.01).

### Gene and accession number

The raw RNAseq data are deposited into CNSA with accession number CNP0007626 (https://db.cngb.org/cnsa/). Gene sequences in woodland strawberry are available from the Dicots PLAZA 5.0 database (https://bioinformatics.psb.ugent.be/plaza) using accession numbers: *FveTRM1*, FvH4_6g46710; *FveTRM4*, FvH4_5g13690; *FveTRM5*, FvH4_2g22810.

## Supplementary Material

Web_Material_uhaf199

## Data Availability

All relevant data can be found within the manuscript and its supplementary materials.
